# Bronchoscopic instillation of amphotericin B is a safe and effective measure to treat pulmonary mycosis

**DOI:** 10.3389/fphar.2023.1167475

**Published:** 2023-06-09

**Authors:** Lei Yang, Changqing Yang, Nansheng Wan, Wei Xie, Yu Tian, Yangbao Xiao, Li Luo, Enguo Chen, Jisong Zhang, Xiaoping Wang, Li Xu, Xingguang Wang, Yunzhi Zhou, Lu Guo, Jun Zou, Xingren Liu, Xuguang Wei, Yubao Wang, Jing Feng

**Affiliations:** ^1^ Department of Respiratory and Critical Care Medicine, Tianjin Medical University General Hospital, Tianjin, China; ^2^ Hunan Chest Hospital, Changsha, Hunan, China; ^3^ Department of Respiratory Therapy, Sir Run Run Shaw Hospital, Hangzhou, Zhejiang, China; ^4^ Shandong Public Health Clinical Center, Jinan, Shandong, China; ^5^ Shandong Provincial Hospital, Jinan, Shandong, China; ^6^ Emergency General Hospital, Beijing, China; ^7^ Sichuan Academy of Medical Sciences and Sichuan Provincial People’s Hospital, Chengdu, Sichuan, China; ^8^ Hebei Provincial Shenzhou Hospital, Hengshui, Hebei, China

**Keywords:** amphotericin B, *Mucor*, *Aspergillus*, fungal infection, intrabronchial

## Abstract

**Background and objectives:** In recent years, there has been a significant increase in the prevalence of pulmonary mycosis disease, and its mortality has increased. There are very few studies on treating pulmonary mycosiss with bronchoscopic instillation of amphotericin B. This study investigated the clinical efficacy and safety of bronchoscopic instillation of amphotericin B for treating pulmonary mycosiss.

**Methods:** This was a multi-centre, retrospective clinical study of 80 patients with pulmonary mycosiss who were treated with bronchoscopic instillation of amphotericin B. The efficacy and safety of this treatment were evaluated.

**Results:** Eighty patients were included {51 males; mean [standard deviation (SD)] age, 46 (15.9) years}. The most common underlying cause was haematological malignancy (73.75%). The mean number of bronchoscopic instillations of amphotericin B was 2.4 (SD 1.5). In terms of treatment success, 58 (72.5%) patients achieved complete or partial changes on imaging after treatment. A total of 62 (77.5%) patients achieved complete or partial changes on imaging and/or local limitation of the mycosis infection. Seventy-six (95%) patients achieved complete or partial changes on imaging and/or local limitation of mycosis infection and/or an immunotherapy time window. The efficacy rates for treatment of *Aspergillus* and *Mucor* infections in terms of the three treatment success criteria described above were 73.81% vs. 63.64%, 80.95% vs. 72.73%, and 92.86% vs. 90.91%, respectively.

**Conclusion:** Bronchoscopic instillation of amphotericin B is safe and effective for treatment of pulmonary mycosiss.

## Introduction

In recent years, the prevalence of pulmonary mycosiss has significantly increased with an increase in the number of immunosuppressed high-risk susceptible groups. Even though early diagnosis of pulmonary mycosiss is possible given the continuous improvements in detection methods, and preventive and empirical treatments are used to treat the disease, the mortality rate is still as high as 50%–90% (Morgan et al., 2005). For the clinical treatment of deep fungal infections, commonly used antifungal drugs include polyenes (amphotericin B), triazoles (fluconazole, voriconazole, itraconazole), and echinocandins (caspofungin) (Walsh et al., 2008).

Pulmonary mycosiss, especially mycotic infections, are associated with the following characteristics: rapid dissemination, requiring timely drug intervention (Walsh et al., 2012); short-term tissue necrosis and local structural destruction of the lung; rapid formation of fibrous and granulation tissue encapsulation after control with effective antifungal drugs; and autoimmune limitation. These characteristics indicate the possible failure of transvenous pharmacologic interventions due to poor local blood flow.

Amphotericin B is a polyene antifungal drug with broad-spectrum effects. When administered intravenously, the drug concentration in the pleural fluid, ascites fluid, and synovial fluid is usually less than half of the blood concentration, and the drug concentration in bronchial secretions is even lower (Stone et al., 2016). However, in some cases, it is the only effective drug for treating acute deep fungal infections; it is highly water-soluble, absorbed less slowly through the airway mucosa, and causes no significant irritation to the airway mucosa. Based on these pharmacological and metabolic characteristics, local instillation of amphotericin B via bronchoscopy has irreplaceable advantages and is worthy of clinical promotion (Lass-Flörl et al., 1998; Polak, 1999).

For some lesions that do not communicate with the bronchi and have little contact with the bloodstream, this makes it difficult for antifungal agents to reach them through the blood. So we need new ways of treating these patients. J L Hargis et al., P Krakówka et al., G BROUET al., and M J Shapiro al. Proposing new treatment modalities (percutaneous instillation of intracavitary amphotericin B) (Brouet et al., 1964; Krakowka et al., 1970; Hargis et al., 1980; Shapiro et al., 1988). Kravitz et al. (2013) retrospectively reviewed 23 patients admitted to our institution for aspergilloma-associated hemoptysis over an 8-year period and underwent percutaneous intracavitary instillation of amphotericin B (ICAB); and identified ICAB as A form of short-term treatment. Takeda et al. (2014); Parikh et al. (2017) respectively, report a case of pulmonary aspergillosis treated with bronchoscopic instillation of amphotericin B. There are few articles on the treatment of such patients above, and the sample size of the published articles is small, or it is a case report or a relatively early study. There are even fewer articles on the bronchoscopic instillation of amphotericin B. There are also variations in the clinical efficacy of bronchoscopic instillation of amphotericin B (Lang et al., 2020). Endoscopic injections have demonstrated good therapeutic effects in multiple case reports and clinical studies (Hargis et al., 1980; Kravitz et al., 2013). However, because the total number of patients is small and most have serious comorbidities, it is difficult to balance the basic conditions among the research groups; it is difficult to implement large-scale multi-centre clinical studies. Therefore, to date, there are no recognised relevant clinical data or specific indicators or parameters for evaluating the benefits to patients.

Accordingly, we designed this “clinical study on the local application of amphotericin B via bronchoscopic instillation in pulmonary lesions” By collecting the clinical data of patients with pulmonary mycosis who met the inclusion criteria and performing statistical analysis, the clinical efficacy and safety of bronchoscopic instillation of amphotericin B in the treatment of pulmonary mycosiss were revealed.

## Methods

### Study design

**FIGURE 1:**
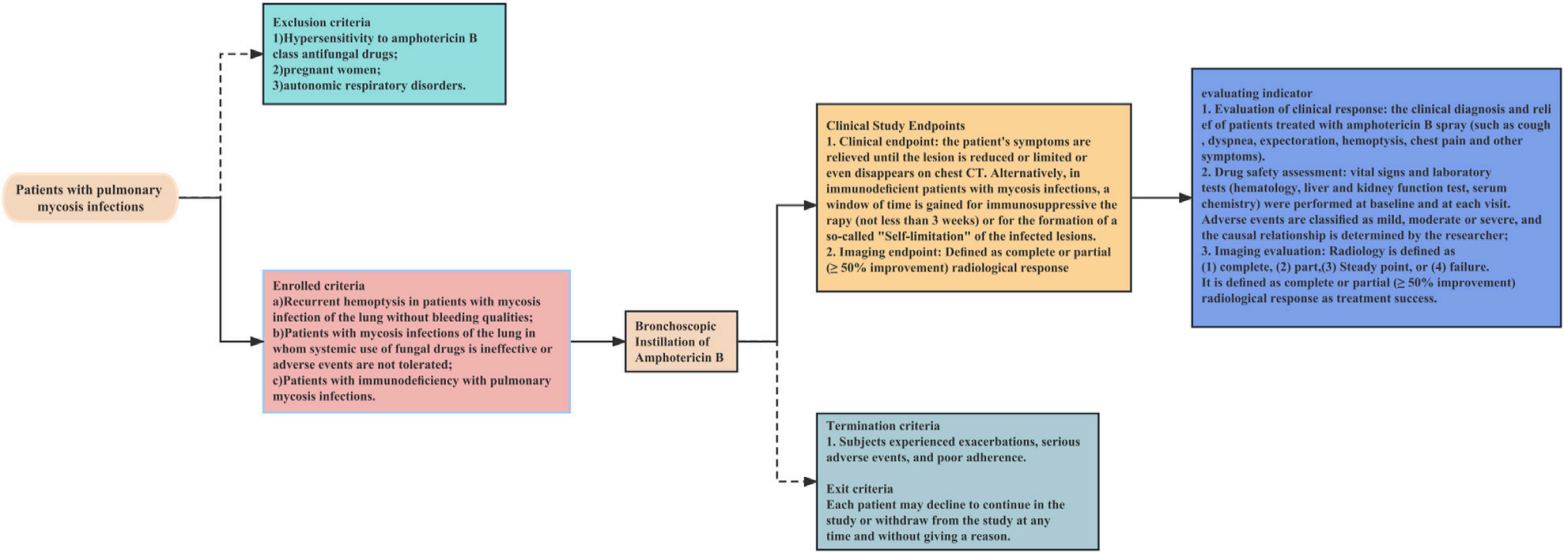
Flow chart of the clinical study of bronchoscopic instillation of amphotericin B.

The operational flow of the whole study is shown in Figure 1. This is a retrospective study of a multicentre clinical treatment trial of patients diagnosed with pulmonary mycosis based on the European Organization for Research and Treatment of Cancer/Mycoses Study Group criteria (Donnelly et al., 2020). The patients enrolled in this study were divided into the following three categories:

1. Recurrent haemoptysis and no bleeding tendency. (If surgical resection was not an option to control recurrent haemoptysis (possible meaning: lesion communicates with the airway, some solution must re-entry into the airway), then in patients without bleeding tendency, bronchoscopic instillation of antifungal drugs may be considered).
2. Systemic use of fungal drugs that was ineffective or involved adverse events that could not be tolerated. Systemic antifungal therapy is considered ineffective if any one or more of these criteria are met (Sanguinetti et al., 2019; Donnelly et al., 2020; Zhang and Zhu, 2020; Alexander et al., 2021; Acet-Öztürk et al., 2022; Fisher et al., 2022). 1) Lack of clinical improvement: the patient does not show any clinical improvement in their symptoms or condition despite receiving systemic antifungal therapy for a sufficient duration of time. 2) Persistence of radiographic abnormalities: radiographic abnormalities, such as pulmonary infiltrates or nodules, persist or worsen despite systemic antifungal therapy. 3) Continuous fungal culture positivity: fungal cultures from respiratory specimens continue to be positive despite systemic antifungal therapy. 4) Lack of serum biomarker response: serum biomarkers, such as galactomannan or beta-D-glucan, do not improve or continue to be positive despite systemic antifungal therapy. 5) Development of antifungal resistance: fungi developed resistance to the systemic antifungal agents.
3. Immunodeficiency. Every immunocompromised patient with pulmonary mycosis should receive treatment, with a few exceptions, especially those with elevated inflammatory markers, such as C-reactive protein. The main indication for endoscopic drug injection is that the patient suffers from the trachea, bronchi and/or pulmonary mycosis, and the pulmonary mycosis lesions have clear localized drainage bronchi on imaging.

The exclusion criteria were:

1. Hypersensitivity to amphotericin B class antifungal drugs
2. Pregnancy
3. Autonomic respiratory disorders

The termination criteria were:

1. Exacerbation of disease
2. Serious adverse events
3. Poor compliance

A decision to withdraw from the study was not subject to potential treatment constraints and did not affect the patient’s medical care. Patients were permitted to withdraw from the study at any time without providing a reason.

### Procedure for bronchoscopic instillation of amphotericin B

**FIGURE 2:**
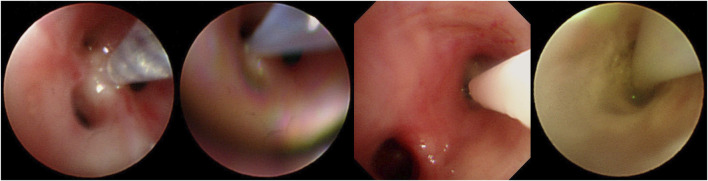
Operation procedure of instilling amphotericin B under bronchoscope.

Before the intrabronchial instillation of the drug, the patient’s condition will be assessed to ensure patient safety. An absolute contraindication to bronchoscopy is severe refractory hypoxia that cannot maintain adequate oxygenation during the procedure due to the patient’s disease (Severe hypoxemia is defined as resting arterial oxygen partial pressure (PaO2) < 60 mmHg or blood oxygen saturation (SpO2) < 90%). After providing written informed consent, all patients were administered amphotericin B via bronchoscopy under local or general anaesthesia. During the instillation operation under the bronchoscope, oxygen inhalation or high-frequency ventilation is given, and the patient’s vital signs are monitored by ECG monitoring. Five ml of water was added to each of the two 5-mg ampoules of amphotericin B deoxycholate and shaken thoroughly to dissolve for injection with a 10-mL syringe. Ten ml of completely dissolved amphotericin B deoxycholate solution was drawn from the two ampoules with a 20-mL syringe, and then 5 mL of air was drawn for propulsion to make a total of 15 mL (10 mL of a solution containing 10 mg of amphotericin B deoxycholate). For bronchoscopic instillation of amphotericin B, the bronchoscope was wedged into the segmental or subsegmental bronchus based on prior computed tomography (CT) thoracic lumen localisation or virtual navigation bronchoscopy; if a definite lesion was visible on bronchoscopy, the bronchoscope was wedged into the lumen. For two focal targets, a total dose of amphotericin B deoxycholate of 10–15 mg was recommended, and for multiple focal targets, the total quantity of amphotericin B deoxycholate was increased to 15–20 mg, and the amount of solvent (water for injection) was increased appropriately, divided into multiple syringes. Precise local injection was carried out visually or using guidance, with ultra-fine endoscopy for each of the target treatment sites. Try to push the bronchoscope front end into the target bronchus or probe into the lesion, and inject it directly; or insert the injection tube or spray tube (such as the unique injection tube or spray tube for bronchoscope) deep into the distal end of the target bronchus, or even directly insert the injection tube, The spray tube is placed in the lesion, and then injected (Figure 2). Each target treatment site received more than 2.5 mg of amphotericin B deoxycholate at each target site, and more than 5 mg of the drug was dispensed at important focal targets. In order to ensure local retention of the drug in the lesion, this can be achieved by using the front end of the endoscope for blocking, with posing and other methods.

### Clinical study endpoints

**FIGURE 3:**
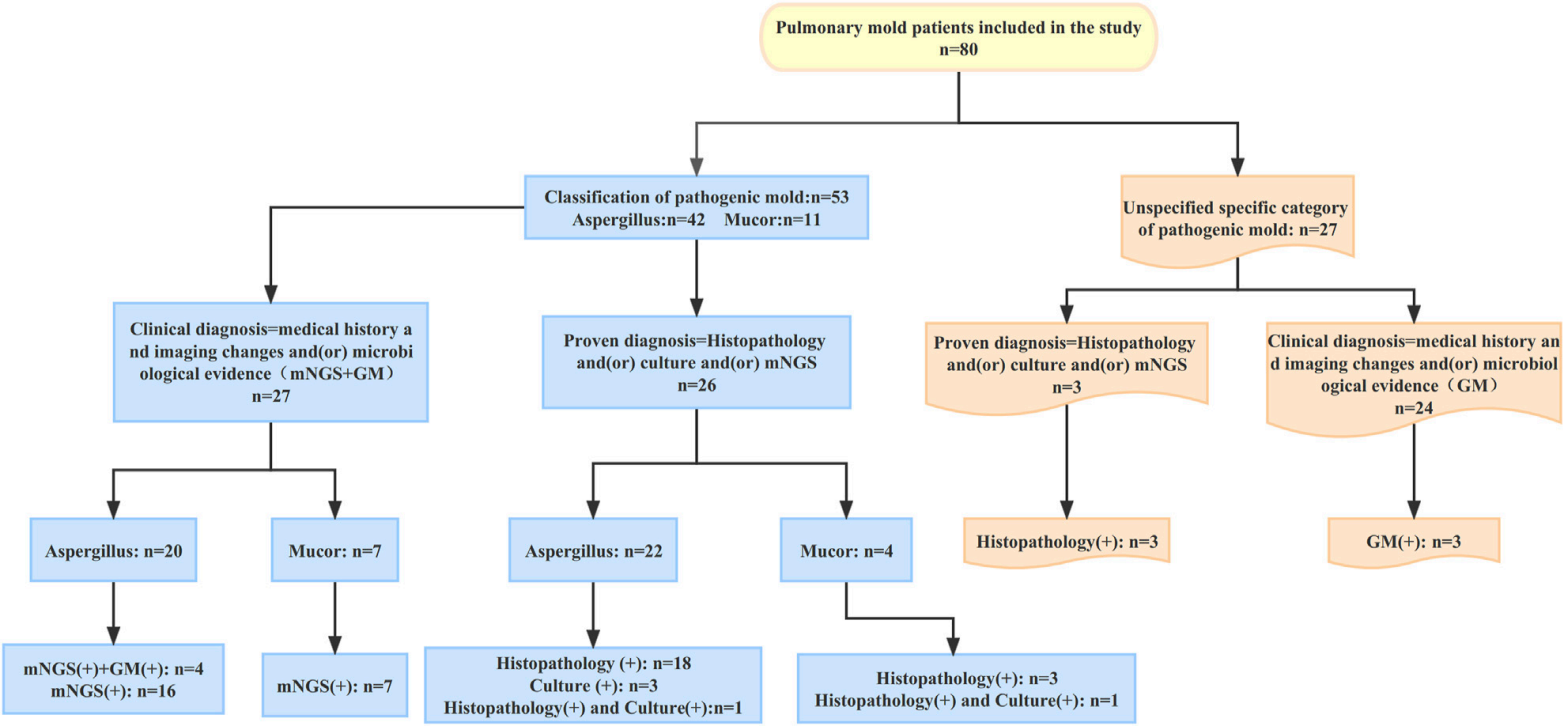
Diagnosis process of 80 patients with pulmonary mold infection Changes in the patient’s chest CT after treatment with intrabronchial amphotericin B instillation.

After initiating amphotericin B treatment via bronchoscopic instillation, the patient’s symptoms were relieved until the lesion was reduced, limited, or disappeared on chest CT. Alternatively, in immunodeficient patients with mycosis infections, a window of time was achieved for immunosuppressive therapy (not less than 3 weeks) or for the development of self-limitation of infected lesions (clearly defined fibrous, mechanised encapsulation around the lesion that has been stable for 3 months) Figure 3.

### Efficacy assessment

CT scans before and after bronchoscopic drug instillation were compared by an independent radiologist who was blinded to the patient information. A radiological response was defined as follows (Cadranel et al., 2012): 1) Complete: complete absorption of the chest CT lesion, 2) Partial: ≥50% reduction in the sum of all measurable lesions on chest tomography, 3) Stable: slight (<50%) or no improvement, or 4) Failure: deterioration of the condition (Cadranel et al., 2012). Treatment success was defined as a complete or partial (≥50% improvement) radiological response, achievement of a window of time (not less than 3 weeks) for immunosuppressive therapy in patients with immunodeficiency with mycosis infections or for the development of self-limitation of infected lesions.

### Statistical analysis

Continuous variables are expressed as mean [SD (standard deviation)]. The discrete variables are expressed as frequency and rate. A histogram was used to compare the differences in the efficacy rates of *Aspergillus* and *Mucor* after instillation treatment.

## Results

### Patient characteristics

The demographics of all patients are shown in Table 1 and Supplementary Table S1. In this multi-centre study, 80 patients underwent bronchoscopic amphotericin B instillation. Of these, 51 (63.75%) were male and the mean (SD) age of the total study population was 46 (15.93) years. In this study, most patients (59/77, 73.75%) had haematological malignancies, five had tuberculosis as their underlying disease, five had diabetes, and two had lung cancer. A total of 58 (72.5%) patients received the systemic antifungal therapy followed by the bronchoscopic instillation of amphotericin B due to ineffectiveness of the systemic antifungal therapy. And the rest 22 patients (27.5%) simultaneously received the systemic antifungal therapy and the bronchoscopic instillation therapy.

**TABLE 1:** Baseline patient characteristics.

Variables	Patients [*n* (%)]
Age (y)	46 ± 15.93
Sex	
Male	51 (63.75%)
Female	29 (36.25%)
Underlying Disease	
Hematological malignancies	59
Tuberculosis	5
Lung cancer	2
Chronic obstructive pulmonary disease	1
Non-tuberculosis mycobacteria	1
Diabetes	5
Others	7
Status of systemic antifungal therapy before instillation of amphotericin B	
Yes	58 (72.5%)
No	22 (27.5%)

According to the EORTC/MSG consensus criteria, all patients had a proven or clinical diagnosis of pulmonary mold infection. The criteria for proven diagnosis are mainly positive histopathology and/or positive culture; the criteria for clinical diagnosis are the medical history and typical imaging changes and/or microbiological evidence (mNGS and GM). In this study, a total of 42 patients with pulmonary Aspergillus infection were identified by histopathology and (or) culture and (or) mNGS to identify the specific pathogenic fungi. Of the 42 patients with pulmonary Aspergillus infection, 22 were proven diagnoses; the other 20 were clinical diagnoses. In 11 patients with pulmonary mucor infection, the specific pathogenic fungi were identified by histopathology and (or) culture and (or) mNGS. Among these 11 patients, four were proven diagnoses; the remaining seven were clinical diagnoses. Except for the above 53 patients whose specific pathogenic fungi were identified, the other 27 mold patients failed to identify the specific pathogenic fungi. Among the 27 patients, 3 were proven diagnoses and 24 were clinical diagnoses.

### Efficacy analyses

The clinical characteristics and operative information of all patients are shown in Table 2. Sixty-three (77.75%) had unilateral involvement. The sites of pulmonary abnormalities on chest HRCT included the right upper lobe for 32 patients, the right middle lobe for 7 patients, the right lower lobe for 10 patients, the left upper lobe for 26 patients, and the left lower lobe for 16 patients. Chest HRCT showed consolidations in 61 patients, nodules in 47 patients, ground-glass opacities in 42 patients, and cavities in 24 patients. The mean number of intrabronchial amphotericin B instillations was 2.4 (1.5). Twenty-seven patients underwent one session, 21 patients underwent two sessions, 12 patients underwent three sessions, 13 patients underwent four sessions, and six patients underwent more than five sessions of intrabronchial amphotericin B instillation. A total of 58 (72.5%) patients achieved complete or partial changes on imaging after bronchoscopic instillation of amphotericin B treatment, 62 (77.5%) patients achieved complete or partial changes on imaging and the formation of self-limitation of infected lesions, and 76 (95%) patients achieved complete or partial specimens in imaging and self-limitation and immunotherapy time windows.

**TABLE 2:** Treatment with bronchoscopic instillation of amphotericin B.

Variables	*n* (%)
Patient’s chest CT features	
Unilateral	63 (77.75)
Bilateral or multiple	17 (21.25)
Lung lobe involvement	
Right upper lobe	32
Right middle lobe	7
Right lower lobe	10
Left upper lobe	26
Left lower lobe	16
Imaging changes	
Consolidation	61
Nodules	47
Ground-glass opacity	42
Cavities	24
No. Sessions of intrabronchial amphotericin B instillation	2.4 ± 1.5
1 session	27
2 sessions	22
3 sessions	12
4 sessions	13
5 sessions	4
6 sessions	2
Successful treatment with intrabronchial amphotericin B instillation Imaging response (Complete + Partial)	58 (72.5%)
Imaging response (Complete + Partial)+ self-limitation	62 (77.5%)
Imaging response (Complete + Partial)+ self-limitation + Immunotherapy time window	76 (95%)

### Typical imaging response

**FIGURE 4:**
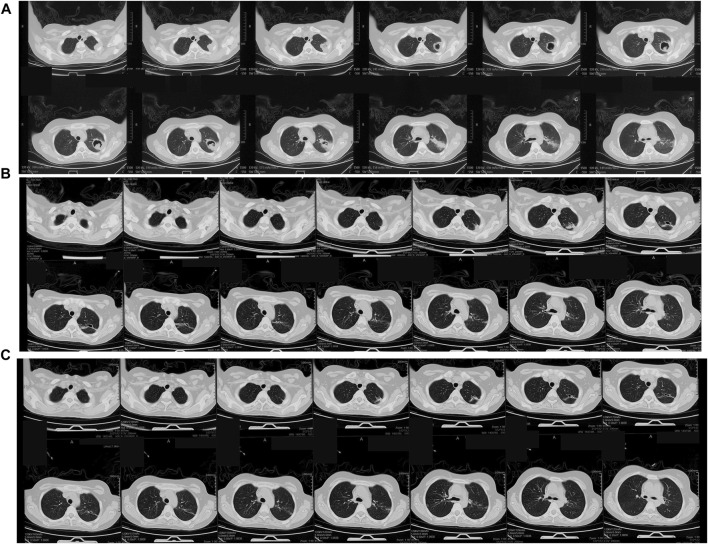
**(A)** Before treatment of bronchoscopic instillation, chest CT shows a solid left upper lobe shadow with cavity formation visible within it. **(B)** 55 days after treatment, chest CT shows that the solid shadow in the upper lobe of the left lung is reduced, and the cavity is further reduced. **(C)** 88 days after treatment, chest CT shows further reduction of solid shadow in the upper lobe of the left lung. Comparison of efficacy between Aspergillus and Mycor patients.

In this study, patient no. 36, who had been diagnosed with acute lymphoblastic leukaemia 3 months prior, was admitted with “intermittent fever for 1 month, cough, and sputum for 1 week”. Before treatment with respiratory endoscopy, chest CT suggested a solid shadow in the upper lobe of the left lung with cavity formation (Figure 4A). After bronchoscopy, a final clinical diagnosis of a pulmonary mycosis was made. After respiratory endoscopy, the patient was treated with amphotericin B (10 mg) in the sub-subsegment of the ascending apical segment of the left upper lobe, posterior segment of the ascending apical segment of the left upper lobe, and posterior segment of the ascending apical segment of the left upper lobe. After two instillations, the patient’s chest CT lesion was significantly more absorbed than before and the patient’s clinical symptoms were significantly relieved (Figures 4B, C).

Evaluation of the efficacy of intrabronchial amphotericin B instillation in the treatment of pulmonary mycosiss caused by different fungal species.

**FIGURE 5:**
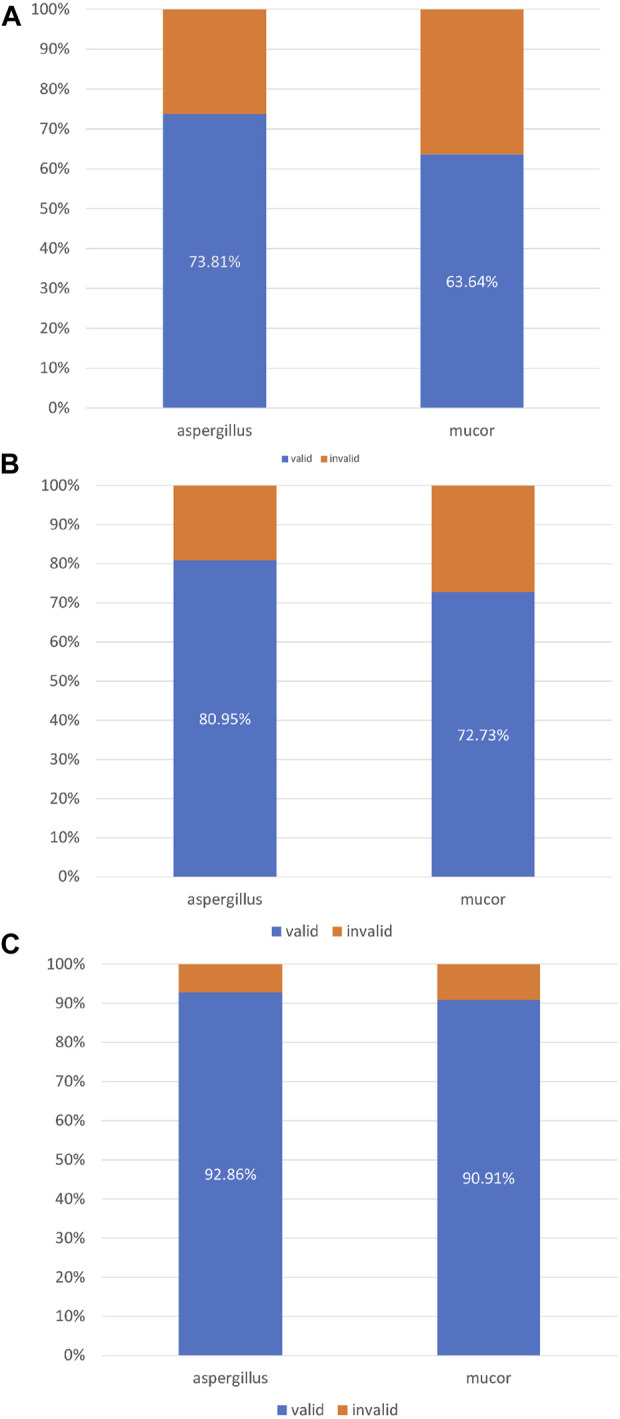
**(A)** Imaging response (complete + partial). **(B)** Imaging response (complete + partial) + self-limitation. **(C)** Imaging response (complete + partial) + self-limitation + immunotherapy time window.

In this study, 42 patients were infected with *Aspergillus* and 11 patients were infected with *Mucor*. A total of 31 patients with *Aspergillus* infection achieved complete or partial improvement on chest imaging after bronchoscopic instillation of amphotericin B. A total of 7 patients with *Mucor* infections achieved complete or partial progress on chest imaging after intrabronchial amphotericin B instillation (Figure 5A). Among the patients with *Aspergillus* infection, 34 patients showed complete or partial improvement and “self-limitation” changes on imaging. Among the patients with *Mucor* infection, eight patients showed complete or partial improvement and “self-limitation” changes on imaging (Figure 5B). Thirty-nine patients with *Aspergillus* infection achieved (complete or partial) improvement on imaging after bronchoscopic instillation, with “self-limitation” and a time window for immunotherapy. Ten patients with *Mucor* infection performed (complete or partial) improvement on imaging after bronchoscopic instillation, “self-limitation” and a time window for immunotherapy (Figure 5C).

### Safety analysis

Seven of the 80 patients (7.5%) experienced adverse reactions, including one patient who developed a sore throat after the first bronchoscopic instillation, which was tolerated by the patient. It was not treated symptomatically and did not require withdrawal from the next bronchoscopic operation. The patient’s sore throat was most likely related to the bronchoscopic operation and was not directly related to the intrabronchial amphotericin B instillation. The patient developed severe dyspnoea after the second bronchoscopic instillation and was intubated and treated symptomatically, and finally, the patient withdrew from the study. Six patients developed drug fever after bronchoscopic instillation and were administered symptomatic treatment; no other symptoms were observed. Of the 80 patients treated with bronchoscopic instillation, none showed any significant deviation from baseline in terms of blood, liver, and kidney function. Eighty patients were treated with bronchoscopic instillation; one had a sore throat, which was considered to be related to the bronchoscopic operation. The remaining patients did not have any complications such as fatal haemoptysis, pneumothorax, arrhythmia, or death.

## Discussion

Pulmonary mycosis spreads rapidly and requires drugs and intervention; In the short term, it can cause tissue necrosis and structural damage to local lung tissues. After corresponding treatment, fibrous and granulation tissue parcels can be formed rapidly. Due to the above characteristics, intravenous drug intervention may hardly achieve the desired clinical outcome due to ischemia. Bronchoscopic instillation of antifungal drugs can provide precise local treatment of target lesions. Studies on the bronchoscopic instillation of antifungal drugs are scarce. Based on animal studies demonstrating the safety of nebulised amphotericin B-administered endobronchial therapy, Ramirez reported the first series of such therapies in humans in 1964 (Lang et al., 2020). The results were impressive, as all three patients showed significant clinical improvement and clearance of the fungal infection. Several subsequent studies also reported success with endobronchial therapy using amphotericin B, ketoconazole, fluconazole, and miconazole (Hamamoto et al., 1983; Yamada et al., 1993; Hinerman et al., 2002). Although all patients showed symptomatic improvement, only six of nine patients showed complete clearance of the fungal balls. Two case reports demonstrated the effectiveness of bronchoscopic instillation of amphotericin B in the treatment of pulmonary mucor and aspergillus infections (Alfageme et al., 2009; Morales et al., 2009). Bronchoscopic instillation of antifungal drugs can effectively control the hemoptysis caused by pulmonary aspergilloma and reduce the severity of hemoptysis caused by inoperable pulmonary aspergilloma (Mohan et al., 2017a; Hadda et al., 2022). The lesions did not change significantly during systemic antifungal therapy alone. However, lesion size was significantly reduced after additional bronchoscopic instillation of amphotericin B (Winkler et al., 2007).

Compared with previous studies, the current study included 80 patients and was multi-centre. Under virtual navigation guidance, injecting drugs with an ultrafine endoscope allows precise local treatment of the target lesions. However, these drugs are not generally used to prevent pulmonary mycosis. Therapeutic drugs can be directly sprayed into the local drainage bronchus of the lesion, in the lesion, or the superior drainage bronchus, forming a long-term high-concentration drug solution environment in or around the target lesion to directly “remove the pathogen”, “reduce the pathogen”, or limit the lesion expansion. Bronchoscopic instillation of amphotericin B can achieve partial or complete remission on imaging or microbiology as well as localisation and stabilisation of lesions. It can also be used to determine a time window for immunosuppressive therapy and to control repeated haemoptysis. Another study included 82 patients. After voriconazole titration, haemoptysis resolved significantly in 25 patients (30.5%) after the first treatment and 52 patients (68.3%) after the second treatment. Transient postoperative cough (*n* = 38, 46.3%) was the most common procedure-related adverse event. Follow-up CT (*n* = 47) showed a 54% reduction in varicose tumour size, whereas 40.4% remained unchanged. The median (IQR) haemoptysis-free period was 12 months (IQR, 9 15.5 months). During a median follow-up of 14.5 months (IQR, 9–18 months), 24 (29.3%) patients showed significant haemoptysis, ultimately concluding that endobronchial voriconazole titration is a safe and effective method for controlling pulmonary aspergilloma hemoptysis (Mohan et al., 2017b). In this study, a total of 58 (72.5%) patients achieved complete or partial changes on imaging after treatment with bronchoscopic instillation of amphotericin B. A total of 62 (77.5%) patients achieved complete or partial modifications on imaging, and/or local limitation of the mycosis infection. A total of 76 (95%) patients achieved complete or partial changes on imaging, and/or local limitation of mycosis infection, and/or achieved an immunotherapy time window. It can also be seen from the above data in this study that bronchoscopic instillation of amphotericin B is an effective way to treat pulmonary mycosiss.

Regarding the effectiveness of bronchoscopic instillation of amphotericin B, we must strictly adhere to the indications. Pulmonary mycosis often forms a wrapped pus cavity in the lesion, and the concentration of antifungal agents in the lung tissue by the systemic administration is usually too low to produce therapeutic effects (Lass-Flörl et al., 1998; Polak, 1999). Therefore, to make sure the accurate bronchoscopic instillation of amphotericin B in the pulmonary lesion is crucial for the effectiveness. In our study, according to the patient’s chest CT positioning or under virtual navigation, the front end of the bronchoscope is inserted deep into the lesion segment or subsegmental bronchus. Amphotericin B is directly injected into the lesion accurately and locally, ensuring the concentration of the drug at the lesion site. The skilled physicians performing the bronchoscopic instillation of amphotericin B are essential for not only safety but also effectiveness.

With the rapid cytological evaluation technology of respiratory endoscopic intervention, clinicians can perform endoscopic drug injection at the same time as the diagnosis of tracheal, bronchial, and/or pulmonary mycosis. Integrating clinical information and pathogenic microorganism test results to make an accurate diagnosis, even for microbial species, can optimise drug selection for systemic treatment. This study compared the efficacy of respiratory endoscopic nebulisation with amphotericin B for the treatment of *Aspergillus* and *Mucor*. The efficacy rates for the three treatment success criteria were 73.81% vs. 63.64%, 80.95% vs. 72.73%, and 92.86% vs. 90.91%.

Concerning the safety of the treatment, first of all, we must strictly adhere to the indications of the patient and evaluate whether the patient has any disease that may cause difficulty in maintaining sufficient oxygenation during the operation before instillation under the bronchoscope. The aerosol inhalation of amphotericin B was used as the main pre-induction, to prevent severe adverse reactions such as allergy and wheezing during instillation. Again, the bronchoscopic instillation of amphotericin B deoxycholate in this study was performed by the experienced physicians. Finally, during the treatment process, some unnecessary invasive operations should be reduced to avoid the unwarranted risks, such as bleeding.

Compared to systemic drugs, the systemic absorption of drugs through respiratory endoscopy is lower, and the side effects are relatively few. And the adverse reactions are also related to the lung lesions of the patients. Before the respiratory endoscopic spray treatment, aerosol inhalation of amphotericin B is generally performed for induction before endoscopic injection. The purpose is to prevent severe adverse reactions such as allergy and wheezing during injection, and to gradually adapt the patient’s airway to amphotericin B. Complications and adverse reactions of endoscopic drug injection include: fever, cough, expectoration, wheezing (other underlying diseases such as asthma and chronic obstructive pulmonary disease should be dealt with separately), chest pain (pleural irritation due to amphotericin B), and a small amount of pneumothorax, more purulent secretion drainage, or allergic reaction (Ramirez, 1964; Ikemoto, 1965; Krakowka et al., 1970; Yamada et al., 1993). In this study, seven out of 80 patients (7.5%) had adverse reactions, one patient withdrew from the study due to obvious dyspnoea, six patients developed fever, and all patients returned to normal after anti-thermal treatment. No fatal haemoptysis, pneumothorax, arrhythmia, death, or other complications occurred.

Compared with the percutaneous intraluminal instillation of antifungal drugs, the endobronchial technique allows frequent examination of local adverse effects or disease progression, avoids the risk of pneumothorax associated with percutaneous needle puncture and allows for a more precise injection of amphotericin B drops into the lesion site with the aid of advanced virtual navigation technology.

Nebulized inhalation has less systemic absorption and toxicity. In a wide range of ventilable areas, including airways and alveoli at all levels, an antifungal drug environment with a concentration higher than the minimum inhibitory concentration can be maintained for a long time, and this can be useful for prevention as well as treatment. However, nebulising inhalation has certain disadvantages. Atomised and inhaled antifungal drugs can only reach areas with acceptable ventilation. Lesions with poor local ventilation, such as consolidation or cavitation, can only limit the progression and expansion of lesions, and direct treatment has a limited effect.

This shows the advantages of bronchoscopic instillation of amphotericin B; however, this treatment method has certain shortcomings. One disadvantage of endoscopic injection is that it is less effective for multiple lesions. However, endoscopic drug injections are invasive. According to the degree of the patient’s condition, the patient will experience pain and poor compliance after drug injection. In addition, owing to the condition of the patient’s lesion, intraoperative and postoperative haemorrhages may also occur. The appearance of the abovementioned changes is also related to the patient’s condition.

Our study has some limitations. First, our study was a retrospective study, and some cases were clinically diagnosed, which may also affect the accuracy of this study. Second, further studies with larger sample sizes are needed to prospectively explore the potential of bronchoscopic instillation of amphotericin B in the treatment of fungal infections of the lung.

In summary, for patients with cavitation (single or multiple), bronchopulmonary fistulas, consolidation, and other target lesions in poorly ventilated areas, a sufficient amount of effective antifungal drug should be administered systemically together with bronchoscopic instillation. And bronchoscopic instillation of amphotericin B is a safe and effective form of treatment.

## Data Availability

The original contributions presented in the study are included in the article/Supplementary Material, further inquiries can be directed to the corresponding authors.

## Abbreviations

CT, computed tomography; SD, standard deviation.
