# Supplementary material for: Bronchoscopic instillation of amphotericin B is a safe and effective measure to treat pulmonary mycosis
Source: Front Pharmacol. 2023 Jun 9;14:1167475. doi: 10.3389/fphar.2023.1167475 (PMC10288024; doi:10.3389/fphar.2023.1167475)
Supplement: Supplementary file 1 [file DataSheet1.PDF]

Patient ID	Sex	Age/yr	Underlying disease	Systemic antifungal medication before intrabronchial amphotericin B instillation	Type of fungi	Kidney disease or liver disease	The time interval between each follow-up (day)	Number of chest CT follow-up	Number of intrabronchial amphotericin B instillation	Imaging response (1 = Complete; 2 = Partial; 3 = Stable; 4 = Failure)	Imaging response (Complete+ Partial)	Imaging response (Complete+ Partial)+ Self-limitation	Imaging response (Complete+ Partial) + Self-limitation+ Immunotherapy time window
1	Male	68	COPD	Yes	Aspergillus fumigatus	No	16/32	2	1	3	Yes	Yes	Yes
2	Female	67	AML	Yes	Aspergillus	No	17	1	1	2	No	Yes	Yes
3	Male	62	Non-Hodgkin lymphoma	Yes	Aaspergillus flavus	No	13/25/23	3	2	2	No	Yes	Yes
4	Male	13	ALL	Yes	Aspergillus fumigatus	No	17/28/31	3	4	4	Yes	Yes	Yes
5	Male	48	AML	Yes	Clinical diagnosis	No	5/41	2	2	3	Yes	Yes	Yes
6	Male	37	AA	Yes	Aspergillus oryzae	No	16/15/25	3	2	3	Yes	Yes	Yes
7	Male	46	ALL	Yes	Clinical diagnosis	No	20/25/30/27	4	5	2	No	No	Yes
8	Male	58	AML	Yes	Aspergillus fumigatus	No	14/27/30	3	2	2	No	No	Yes
9	Male	44	AML	Yes	Mycor	No	21/28/26	3	3	3	Yes	Yes	Yes
10	Female	56	AML	Yes	Clinical diagnosis	No	15/29/25	3	1	3	Yes	Yes	Yes
11	Male	53	AML	Yes	Aspergillus fumigatus	No	10/25/32	3	2	3	Yes	Yes	Yes
12	Male	49	AML	Yes	Clinical diagnosis	No	18/28/25	3	1	3	Yes	Yes	Yes
13	Male	41	AML	Yes	Aspergillus flavus	No	14/27/25	3	1	3	Yes	Yes	Yes
14	Female	69	Autoimmune hemolytic anemia	Yes	Clinical diagnosis	Yes	15/32	2	1	3	Yes	Yes	Yes
15	Male	43	AML	Yes	Aspergillus	No	15/25/30	3	3	4	Yes	Yes	Yes
16	Male	65	Autoimmune hemolytic anemia	Yes	Aspergillus flavus	No	12/30/25	2	3	2	No	No	No
17	Female	63	AML	Yes	Aspergillus fumigatus	No	20/29/33	2	1	3	Yes	Yes	Yes
18	Male	61	Diabetes mellitus	Yes	Aspergillus fumigatus	No	30	2	1	3	Yes	Yes	Yes
19	Female	31	AML	Yes	Aspergillus	No	14/28/26/30	4	1	3	Yes	Yes	Yes
20	Female	66	MDS	No	Clinical diagnosis	No	14/29	2	3	2	No	No	Yes
21	Male	33	AML	Yes	Aspergillus	No	17/23	2	2	3	Yes	Yes	Yes
22	Male	16	AML	Yes	Aspergillus flavus	No	16/27/32	3	2	2	No	No	Yes
23	Male	52	AML	Yes	Aspergillus fumigatus	No	20/23/23	3	1	2	No	Yes	Yes
24	Male	36	ALL	Yes	Mycor	No	13/25/23	3	2	2	No	No	Yes
25	Male	30	MDS	Yes	Aspergillus fumigatus	No	16/23	2	1	3	Yes	Yes	Yes
26	Male	58	1	Yes	Mycor	No	13/23/27/32	4	2	2	No	No	Yes
27	Male	47	AML	Yes	Aspergillus flavus	No	17/25/31	3	2	2	No	No	No
28	Female	66	Pulmonary tumor	Yes	Aspergillus fumigatus	No	23/25	2	1	3	Yes	Yes	Yes
29	Female	27	Acute leukemia	Yes	Aspergillus fumigatus	No	16/17/15	3	3	2	No	No	Yes
30	Female	15	AML	Yes	Aspergillus	No	15/24/30	3	1	3	Yes	Yes	Yes
31	Male	14	AA	Yes	Clinical diagnosis	No	18/26	2	1	3	Yes	Yes	Yes
32	Male	40	ALL	Yes	Mycor	No	15/27/31	3	5	2	No	Yes	Yes
33	Female	49	AML	No	Clinical diagnosis	Yes	13/32	2	4	2	No	No	Yes
34	Male	56	ALL	No	Clinical diagnosis	No	17/31/28	3	1	3	Yes	Yes	Yes
35	Female	44	AML	Yes	Mycor	No	12/28/31	3	1	3	Yes	Yes	Yes
36	Female	24	ALL	Yes	Aspergillus flavus	No	15/25/32	3	4	4	Yes	Yes	Yes
37	Female	52	AML	Yes	Clinical diagnosis	No	15/31	2	2	3	Yes	Yes	Yes
38	Male	47	ALL	Yes	Aspergillus flavus	No	17/32/26	3	2	3	Yes	Yes	Yes
39	Male	51	AML	Yes	Aspergillus fumigatus	No	20/25	2	2	4	Yes	Yes	Yes
40	Male	45	AML	Yes	Clinical diagnosis	No	16/32	2	4	3	Yes	Yes	Yes
41	Male	71	Acute leukemia	Yes	Clinical diagnosis	No	12/30	2	1	3	Yes	Yes	Yes
42	Male	47	AA	Yes	Mycor	No	16/29	2	3	2	No	No	No
43	Male	45	AML	Yes	Aspergillus fumigatus	No	13/27/28	3	1	3	Yes	Yes	Yes
44	Female	20	AA	Yes	Proven diagnosis	No	16/29	2	1	3	Yes	Yes	Yes
45	Male	32	AML	Yes	Aspergillus flavus	No	14/26/28	3	1	3	Yes	Yes	Yes
46	Female	44	Diseases of blood system	Yes	Clinical diagnosis	No	15	1	4	3	Yes	Yes	Yes
47	Male	25	Acute leukemia	Yes	Aspergillus oryzae	No	17/28/32	3	3	3	Yes	Yes	Yes

Patient ID	Sex	Age/yr	Underlying disease	Systemic antifungal medication before intrabronchial amphotericin B instillation	Type of fungi	Kidney disease or liver disease	The time interval between each follow-up (day)	Number of chest CT follow-up	Number of intrabronchial amphotericin B instillation	Imaging response (1 = Complete; 2 = Partial; 3 = Stable; 4 = Failure)	Imaging response (Complete+ Partial)	Imaging response (Complete+ Partial)+ Self-limitation	Imaging response (Complete+ Partial) + Self-limitation+ Immunotherapy time window
48	Female	17	Paroxysmal hemoglobinuria	Yes	Mycor	No	14/27/32	3	1	3	Yes	Yes	Yes
49	Male	39	AML	Yes	Aspergillus flavus	No	11/30	2	1	3	Yes	Yes	Yes
50	Female	27	AML	Yes	Aspergillus	No	14/28/27	3	4	4	Yes	Yes	Yes
51	Male	55	AA	Yes	Clinical diagnosis	No	16/28/32	3	3	3	Yes	Yes	Yes
52	Male	45	AML	Yes	Mycor	No	13/28	2	1	3	Yes	Yes	Yes
53	Female	35	AML	No	Aspergillus fumigatus	No	15/29/27/25	4	2	2	No	No	Yes
54	Female	39	Diseases of blood system	No	Aspergillus	No	13/27/32	3	1	3	Yes	Yes	Yes
55	Male	55	MDS	Yes	Clinical diagnosis	No	15/29	2	2	3	Yes	Yes	Yes
56	Female	56	Pulmonary tuberculosis	No	Aspergillus	No	19	1	5	3	Yes	Yes	Yes
57	Male	69	Pulmonary tuberculosis	No	Clinical diagnosis	No	24	1	2	3	Yes	Yes	Yes
58	Female	50	Pulmonary tuberculosis	No	Aspergillus	No	139	1	1	3	Yes	Yes	Yes
59	Male	44	Diabetes mellitus	Yes	Mycor	No	44/26	2	7	4	Yes	Yes	Yes
60	Male	32	No	Yes	Aspergillus flavus	No	14/34	2	4	3	Yes	Yes	Yes
61	Male	53	Diabetes mellitus	Yes	Mycor	No	25/96/71	3	4	3	Yes	Yes	Yes
62	Female	69	Diabetes mellitus	No	Mycor	No	28/34/55	3	8	3	Yes	Yes	Yes
63	Male	57	Pulmonary tumor	Yes	Aspergillus	No	12/13	2	3	1	No	No	No
64	Female	35	No	No	Aspergillus	No	28	1	5	3	Yes	Yes	Yes
65	Male	25	ALL	No	Aspergillus	No	15/27	2	2	2	No	No	Yes
66	Female	70	Hypertension	No	Clinical diagnosis	No	74	1	4	3	Yes	Yes	Yes
67	Male	39	Pulmonary tuberculosis	No	Clinical diagnosis	No	167	1	2	3	Yes	Yes	Yes
68	Male	68	Hypertension	Yes	Clinical diagnosis	No	23/30	2	1	3	Yes	Yes	Yes
69	Male	45	No	Yes	Clinical diagnosis	No	106	1	4	3	Yes	Yes	Yes
70	Male	77	Pulmonary tumor	No	Clinical diagnosis	No	49	1	2	3	Yes	Yes	Yes
71	Male	48	No	No	Aspergillus	No	245	1	1	3	Yes	Yes	Yes
72	Male	52	COPD	No	Aspergillus	No	21/46	2	4	3	Yes	Yes	Yes
73	Female	21	AA	Yes	Clinical diagnosis	No	15/32/34	3	2	2	No	No	Yes
74	Male	17	Diseases of blood system	Yes	Clinical diagnosis	No	21	1	3	2	No	No	Yes
75	Female	41	Acute leukemia	No	Clinical diagnosis	No	84	1	3	1	No	No	Yes
76	Female	54	AML	No	Clinical diagnosis	No	16/25/23	3	2	2	No	No	Yes
77	Male	70	Pulmonary tuberculosis	No	Clinical diagnosis	No	51/53/81	3	4	3	Yes	Yes	Yes
78	Male	55	Nontuberculous mycobacteria	No	Aspergillus fumigatus	No	8/22	2	3	3	Yes	Yes	Yes
79	Female	60	Diabetes mellitus	No	Aaspergillus flavus	No	12	1	2	3	Yes	Yes	Yes
80	Female	57	No	No	Aspergillus	No	42	1	4	3	Yes	Yes	Yes

Abbreviations: AML acute myeloid leukemia, ALL acute lymphocytic leukemia, MDS myelodysplastic syndrome, AA aplastic anemia, COPD chronic obstructive pulmonary disease.
